# Microbial seafood safety assessment following a marine mucilage disaster in the Sea of Marmara

**DOI:** 10.1111/1758-2229.70050

**Published:** 2025-01-23

**Authors:** Didem Üçok, Şehnaz Yasemin Tosun, Nuray Erkan, İdil Can Tunçelli, Hande Doğruyol, Şafak Ulusoy, Sühendan Mol, Özkan Özden, Eda Dagsuyu, Refiye Yanardag

**Affiliations:** ^1^ Faculty of Aquatic Sciences, Department of Fisheries and Seafood Processing Technology, Seafood Processing Programme Istanbul University Istanbul Türkiye; ^2^ Faculty of Aquatic Sciences, Department of Fisheries and Seafood Processing Technology, Food Safety Programme Istanbul University Istanbul Türkiye; ^3^ Faculty of Engineering, Department of Chemistry Istanbul University‐Cerrahpaşa Istanbul Türkiye

## Abstract

Marine mucilage disasters, primarily caused by global warming and marine pollution, threaten food security and the sustainability of marine food resources. This study assessed the microbial risks to public health in common sole, deep‐water rose shrimp, European anchovy, Atlantic horse mackerel and Mediterranean mussel following the mucilage disaster in the Sea of Marmara in 2021. The total viable count, total Enterobacteriaceae count and the presence of *Escherichia coli* O157:H7, *Salmonella* spp., *Listeria monocytogenes*, *Vibrio parahaemolyticus*, *Vibrio cholerae*, *Aeromonas hydrophila*, *Clostridium perfringens* and *Bacillus cereus* were studied during the 2021–2022 fishing season. In September, the first month of the catching season, pathogens in all seafood were markedly higher compared to the entire season: *E. coli* O157:H7: 86%, *Salmonella* spp.: 30%, *L. monocytogenes*: 21%, *V. parahaemolyticus*: 53% and *A. hydrophila*: 100%. The main factors for the high prevalence of pathogens in September are elevated seawater temperature and the fact that it is the first month following the mucilage disaster. Following natural disasters such as mucilage, evisceration of seafood, washing with clean water, depuration of shellfish and ensuring adequate cooking time and temperature are essential for food safety and public health.

## Graphical abstract

This study investigates pathogen contamination in fish species affected by marine mucilage in the Sea of Marmara. Key pathogens such as *Escherichia coli* O157, *Salmonella* spp. and *Listeria monocytogenes* were detected. The data highlight seasonal variations in positivity rates, revealing significant food safety risks associated with marine mucilage.

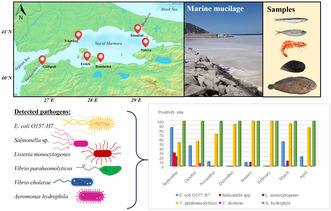

## INTRODUCTION

The dominant power of anthropological effects manifests itself in global warming and environmental pollution. Increasing pollution causes environmental disasters and poses a significant threat to water resources. Effective management of water resources is critical to the sustainability of marine ecosystems. In the context of the United Nations (UN) Sustainable Development Goals (SDGs), the significance of these issues is underscored, particularly in relation to Goal 14 (Life Below Water), Goal 12 (Responsible Consumption and Production) and Goal 13 (Climate Action). Habitat degradation, climate change due to rising temperatures and increased carbon dioxide levels, ocean acidification, algal blooms and chemical and microbiological pollution significantly affect the safety and sustainability of marine‐derived foods both regionally and globally (Erkan, 2023). Marine mucilage, which has increased in frequency in recent years, is a multifaceted danger that negatively affects the life of aquatic organisms, threatens the safety of marine food and may adversely affect human health due to seafood consumption (Dagsuyu et al., 2024; Dogruyol et al., 2024). Marine mucilage, first observed in the Sea of Marmara in 2007, emerged as a large‐scale and rare environmental disaster in 2021. Intensive fieldwork was carried out for approximately 1 month, and 11,084 m^3^ of mucilage aggregates were collected from the sea surface (Yağcı et al., 2022). Kavzoğlu and Goral (2022) study results showed that mucilage aggregates were separated by covering approximately 6 km^2^ of the sea surface on May 14, reached the highest level on May 24 and decreased at the end of July. Mucilage spreading over such a wide area has negatively affected sustainable food security in the Sea of Marmara, the second most productive sea in Türkiye. This sea has been exposed to intense pollution for many years without any precautions. With the addition of poorly managed environmental and fisheries policies, this environmental disaster has been inevitable (Erkan, 2023). This case of mucilage, which is rarely seen in the world at this intensity, has raised public health and food safety issues that may arise from seafood consumption. Mucilage is a mucus secreted by phytoplankton in a stressful environment due to intense pollution and also provides a suitable breeding ground for pathogenic microorganisms (Öncül & Aktaş, 2023). The high bacterial load in the mucilage, as well as the number of viruses and prokaryotes, is significantly higher than values typically reported for seawater (Danovaro et al., 2009; Del Negro et al., 2005; Simon et al., 2002) which could potentially affect human health and well‐being in both the short and long term (Öncül & Aktaş, 2023; Yentur et al., 2013).

Mucilage pollution is a major threat to the marine environment that has attracted the attention of the scientific and public communities. Although there is ample evidence that mucilage causes significant harm to the marine ecosystem, particularly to the benthic community and fish larvae, the consumption of marine foods exposed to mucilage and the potential harms are not clear (Demirel et al., 2023; Karakulak et al., 2023).

Fish and other seafood contain valuable nutrients for the human diet and are generally safe for consumption. However, they may pose health risks due to contamination of their habitats or rare but large‐impact environmental disasters (Mol & Cosansu, 2022).

The Sea of Marmara is an inland sea located between the Black Sea and the Aegean Sea. It provides a significant portion of seafood supply to domestic and foreign markets. However, being surrounded by a large population and major industrial areas, it carries a high risk of pollution. This study aimed to determine the microbial risks that may endanger public health in seafood following the mucilage disaster in the Sea of Marmara in 2021. To achieve this, the incidence of pathogens in the most popular seafood species was examined during the commercial fishing season after the mucilage event.

## EXPERIMENTAL PROCEDURES

### Sampling plan

**FIGURE 1:**
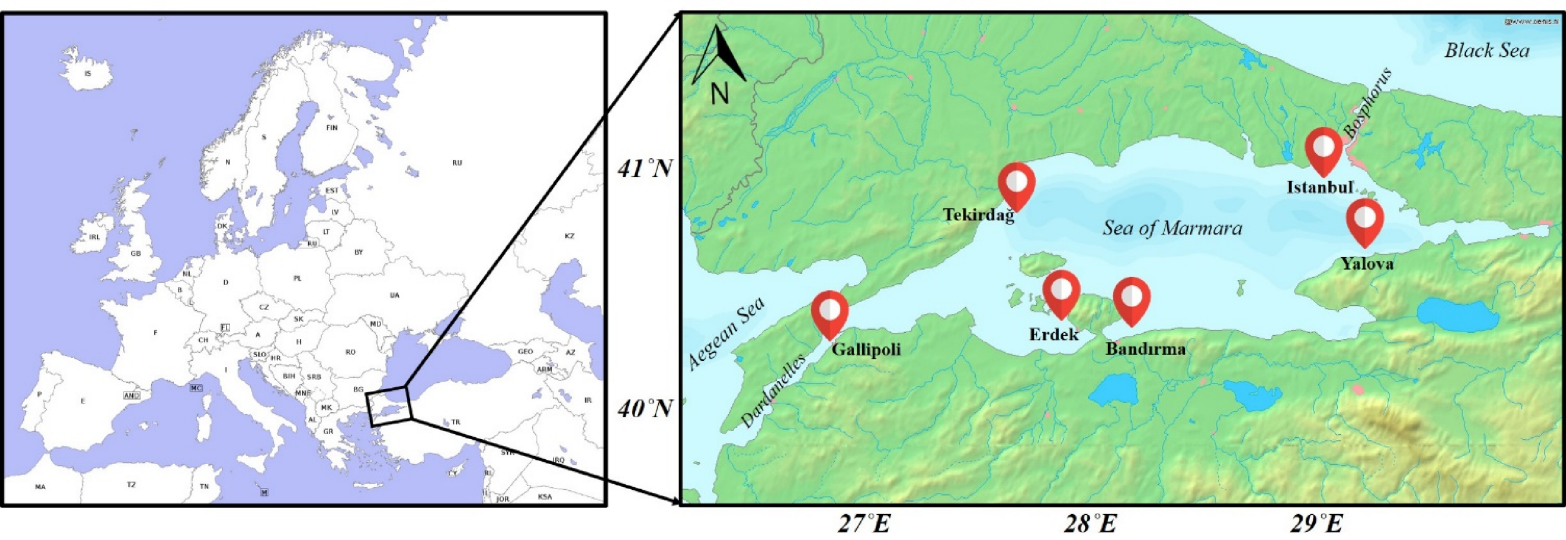
Sampling sites in the Sea of Marmara.

The sampling of the study was carried out from the Sea of Marmara during the 2021–2022 catching season (September 2021–April 2022), following the 2021 mucilage disaster. The commercial fishing season has been established by the ‘Regulation on Commercial Fishing in the Seas and Inland Waters’ issued by the Republic of Türkiye, Ministry of Agriculture and Forestry. During the study, 488 individuals of European anchovy (*Engraulis encrasicolus*, Linnaeus, 1758) and 480 individuals of Atlantic horse mackerel (*Trachurus trachurus*, Linnaeus, 1758) were collected from commercial vessels, fishing in the Sea of Marmara. Ninety‐five individuals of common sole (*Solea solea*, Linnaeus, 1758) were collected from the commercial fishing areas (Tekirdağ, İstanbul and Bandırma) in the Sea of Marmara. Deep‐water rose shrimp (*Parapenaeus longirostris* Lucas, 1846) samples (2044 individuals) were collected from shrimp hunting areas of Tekirdağ, İstanbul, Bandırma and Yalova in the Sea of Marmara. In the study, 507 individuals of wild Mediterranean mussels (*Mytilus galloprovincialis*, Lamarck, 1819) were collected from Bosporus and Dardanelles, and 555 individuals of cultured mussels were collected from farms in Bandırma and Gallipoli. None of the mussels were depurated to determine neutral contamination levels. The sampling areas are shown in Figure 1.

### Microbiological analyses

Samples were brought to the laboratory in foam boxes under cold chain conditions. For total viable count (TVC), total *Enterobacteriaceae* count (TEC), *Clostridium perfringens* count (CPC) and *Bacillus cereus* count, 25 g of flesh samples from anchovy, horse mackerel, sole and shrimp were weighed separately, and for mussels, 25 g of flesh and intravalvular liquid samples were weighed under aseptic conditions and transferred to sterile stomacher bags. Then, 225 mL Maximum Recovery Diluent (MRD) was added and homogenized in the stomacher (Masticator, IUL Instruments, Spain). Following this process, serial dilutions (1:9) were prepared. The total viable count was determined on plate count agar (PCA) medium by the pour plate method. The colonies formed after 24 h of incubation at 37°C were counted, and the results were evaluated (Baumgard, 1986). For the total *Enterobacteriaceae* count, the double‐layer method was applied in the violet red bile dextrose (VRBD) medium. After 18–24 h of aerobic incubation at 35–37°, dark red colonies were counted. The oxidase test was applied to three randomly selected colonies from each petri dish (Halkman, 2019; Paulsen et al., 2008). *Bacillus cereus* count was determined on mannitol egg yolk polymyxin (MYP) agar with the spread plate method and incubated 24–48 h at 28–30°C (Tallent et al., 2020). In order to determine the number of *C. perfringens*, the double‐layer method was applied on a supplemented tryptose sulfite cycloserine (TSC) medium. Anaerobic incubation was performed at 37°C for 24 h using a CO₂ incubator (CO_2_ incubator, Heal Force, Shanghai, Lishen Scientific Equipment Co, Ltd.). At the end of the incubation, typical black colonies were counted (Rhodehamel & Harmon, 2001).

*Salmonella* presence/absence analyses were performed according to Andrews et al. (2024). For this purpose, 25 g of samples was pre‐enriched with 225 mL of buffered peptone water at 37°C for 24 h. Then, 0.1 mL of it was added into Rappaport‐Vassiliadis Broth (10 mL) for enrichment. Test tubes were incubated at 42°C for 24 h. At the same time, a 1 mL sample taken from pre‐enrichment was inoculated into Tetrathionate Broth (10 mL) and incubated at 43°C for 24 h. After incubation, a loopful from each medium (Rappaport‐Vassiliadis Broth and Tetrathionate Broth) was taken and plated on XLT4 agar medium prepared with a selective supplement. Identification tests were performed on suspected *Salmonella* colonies after 18–24 h of incubation at 37°C. For purification, three or five suspected *Salmonella* colonies from each Petri dish were streaked on tryptic soy agar (TSA) and incubated at 37°C for 24 h. After incubation, a loopful of a colony from TSA medium was taken and streaked on Triple Sugar Iron (TSI), and another loopful was streaked on lysine iron (LI) agar. The slant agars were incubated at 35°C for 48 h (TSI Agar) and at 35°C for 24 h (LI Agar). At the end of incubation, additional biochemical tests were performed on suspicious colonies for confirmation.

For the determination of *E. coli* O157:H7, 25 g of sample was incubated with 225 mL of modified EC broth at 35–37°C for 18–24 h. At the end of the incubation, a 0.1 mL sample taken from the enrichment medium was streaked on the surface of sorbitol McConkey agar medium prepared by adding cefixime tellurite selective additive and incubated at 37°C for 24 h. Identification tests were performed on suspicious colourless transparent (sorbitol negative) colonies. Latex agglutination test kits for *E. coli* O157 (Microgen) and H antigen tests (Microgen *E. coli* H7 typing serum) were used for rapid confirmation (Cho et al., 2016; Feng et al., 2018).

For detection of *L. monocytogenes*, 25 g of sample was incubated with 225 mL of Buffered Listeria Enrichment Broth at 30°C for 4 h, and then, Listeria selective enrichment supplement was added. After 44 h of incubation at 30°C, a 0.1 mL sample was taken from the enrichment medium and inoculated onto Palcam Listeria selective agar, which had been prepared with Palcam Listeria selective supplement. The spread plate method was used for inoculation, and the plates were incubated at 35°C for 24–48 h. At the end of incubation, 1.5–2 mm diameter, black‐zoned, olive green colonies were subjected to biochemical and identification tests for confirmation (Hitchins et al., 2022).

The presence/absence of *V. parahaemolyticus* and *V. cholerae* in anchovy and horse mackerel was determined according to Kaysner et al. (2019). For the enrichment, 25 g of the sample was incubated with 225 mL of alkaline peptone water (APW) at 35°C for 24 h. Then, a loopful was streaked onto thiosulfate‐citrate‐bile salt sucrose (TCBS) medium and incubated at 35°C for 18–24 h and onto HiChrom Vibrio Agar, which was incubated at 35–37°C for 18–24 h. Biochemical tests were conducted on suspected colonies for identification. In TCBS medium, small yellow colonies were identified as *V. cholerae*, while small colonies with blue‐green centres were identified as *V. parahaemolyticus*. On HiChrom Vibrio Agar, bluish‐green colonies indicated *V. parahaemolyticus*, whereas purple colonies were identified as *V. cholerae*.

For the detection of *A. hydrophila*, 25 g of sample were homogenized with 225 mL alkaline peptone water (0.1%) and incubated at 37°C for 24 h. After the enrichment, a loopful sample was streaked on aeromonas selective agar, incubated at 35–37°C for 18–24 h, and a confirmation test was performed on translucent colonies (Lee et al., 2021; Üçok et al., 2023).

### Statistical analyses

Statistical analyses were performed using the IBM SPSS Statistics 20 software program. The differences, depending on the months, were compared via one‐way ANOVA. Differences between demersal‐benthic and pelagic species were determined by *t*‐test. Results are given as means and standard deviations. The results were evaluated at the *p* <0.05 significance level.

## RESULTS AND DISCUSSION

Following the 2021 mucilage disaster, a total of 4169 seafood samples were analysed during the commercial fishing season (September 2021–April 2022), and 9324 pathogens were isolated. During the catching season, the proportions of pathogenic bacteria detected in these samples were as follows; *E. coli* O157:H7: 32.69% (1363), *Salmonella* spp.: 5.61% (234), *L. monocytogenes*: 6.06% (253), *V. parahaemolyticus*: 78.43% (3270), *V. cholerae*: 0.83% (35) and *A. hydrophila*: 100% (4169). Yılmaz et al. (2005) examined *Mytilus galloprovincialis* and *Venus gallina* collected from the Sea of Marmara and reported that they did not detect *Vibrio* species or *Salmonella* spp. in any of the samples. Similarly, in a study conducted by Arık Çolakoğlu et al. (2010), *V. parahaemolyticus* was detected in only two out of five stations, and *Salmonella* was absent in all stations. This contrasts with our findings, where *V. parahaemolyticus* was present in 78.43% of seafood samples throughout the fishing season. While *Salmonella* spp. was not detected in these studies, it was found in our study during the first sampling conducted immediately after the mucilage disaster. Additionally, Altuğ and Güler (2002) detected varying loads of *E. coli* each month in their six‐month analysis of sea snails collected from the Sea of Marmara. In our study, *E. coli* O157:H7 was also commonly detected. Prior to the mucilage disaster, the presence of *E. coli* was found in mussel samples collected from similar stations in the Dardanelles. The impact of the mucilage disaster on the microbial contamination of seafood is likely due to the role of mucilage as a good reservoir for microbial growth.

**FIGURE 2:**
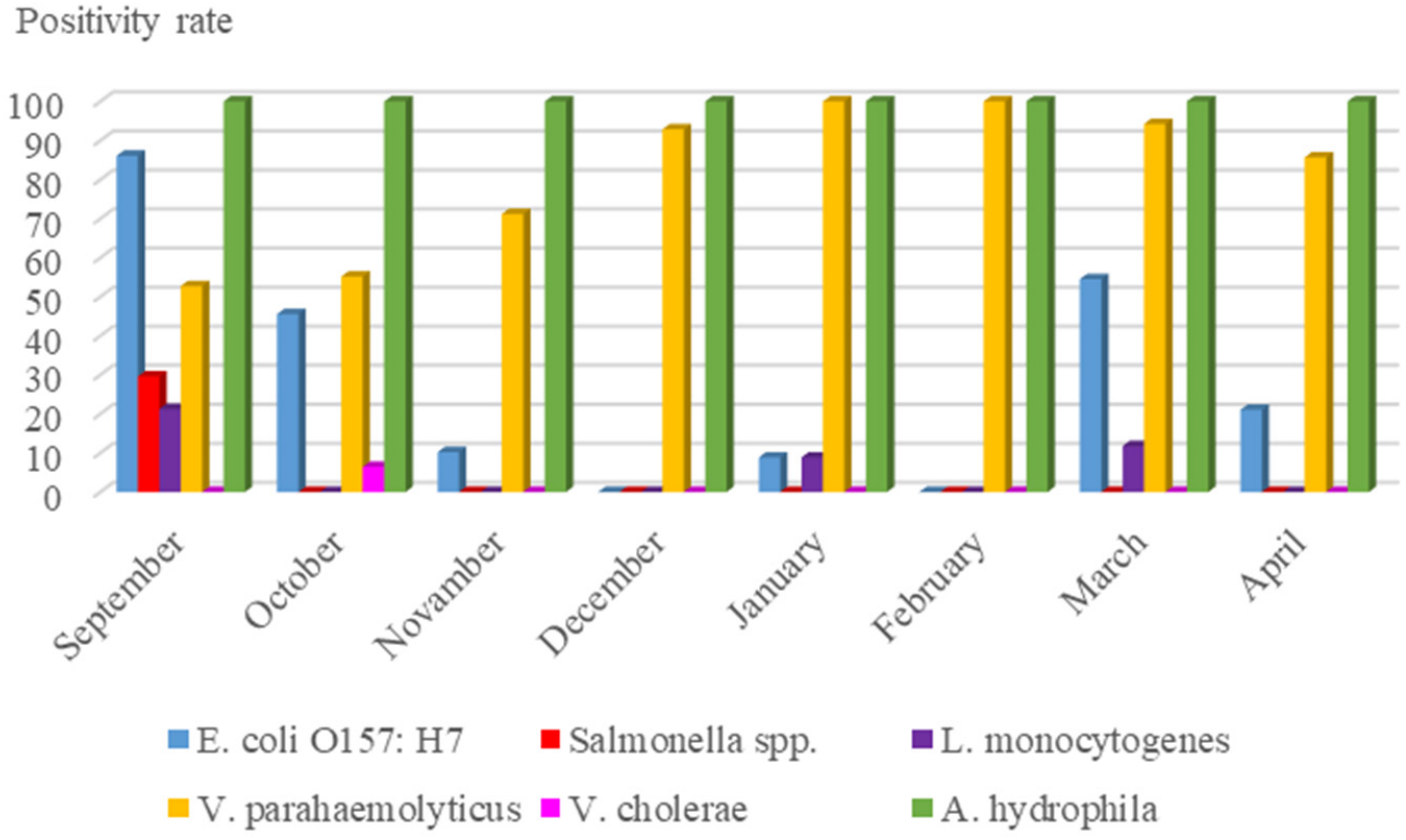
The detection rates of six foodborne pathogenic bacteria according to months.

In September, the proportion of pathogens in all seafood species was found to be markedly higher compared to the entire fishing season, as shown below: *E. coli* O157:H7: 86%, *Salmonella* spp.: 30%, *L. monocytogenes*: 21%, *V. parahaemolyticus*: 53% and *A. hydrophila*: 100% (Figure 2). September is the first month of the fishing season following the mucilage disaster, and the sea water is still warm. These are the main factors for the high incidence of pathogens in September compared to the rest of the fishing season.

**FIGURE 3:**
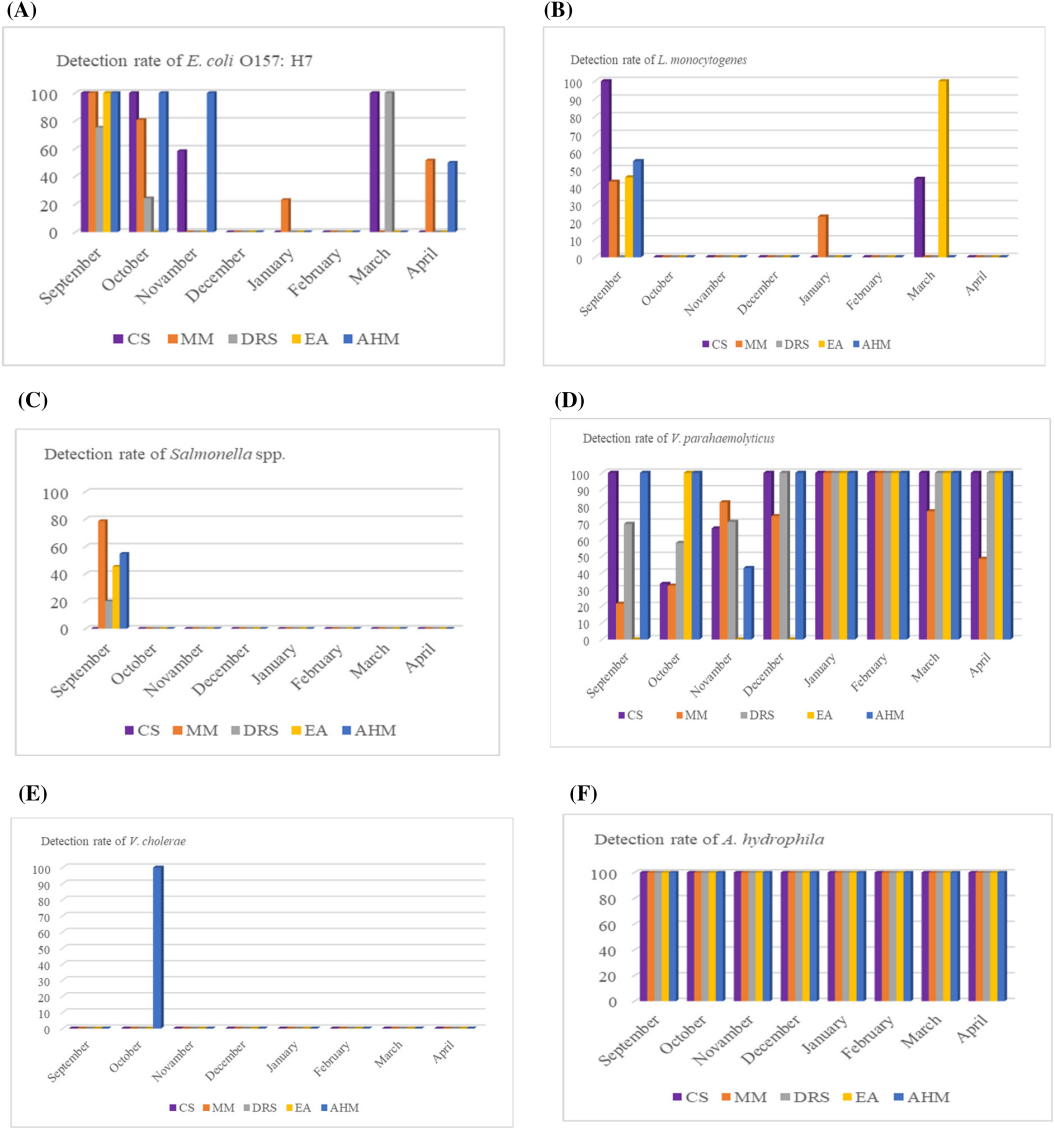
Detection rate of *E. coli* O157:H7 (A), *L. monocytogenes* (B), *Salmonella* spp. (C), *V*. *parahaemolyticus* (D), *V. cholerae* (E) and *A*. *hydrophila* (F) in seafood according to months. AHM, Atlantic Horse Mackerel; CS, Common Sole; DRS, Deep‐water Rose Shrimp; EA, European Anchovy; MM, Mediterranean Mussel.

The natural habitat of *E. coli* and *Salmonella*, the most important members of the *Enterobacteriaceae* family, is usually animals and humans. These bacteria can be transmitted to seafood during handling and processing or from their polluted aquatic habitat. The primary sources of *E. coli* and *Salmonella* transmission to water sources and the marine environment are untreated urban sewage, along with wild and domestic animals (Amagliani et al., 2012; Gram, 2003). Polluted seawater sediment can be a good reservoir for *E. coli* (Gerba & Mcleod, 1976). Altuğ et al. (2013) pointed out that the abundance of *Enterobacteriaceae* members, including *Salmonella enterica* and *E. coli*, are indicators of anthropogenic pollution input in the Sea of Marmara. Mucilage is a good habitat for microorganisms such as bacteria and viruses (Danovaro et al., 2009; Del Negro et al., 2005). Altuğ et al. (2021) detected total faecal coliform and intestinal streptococcus levels above the limit values in the samples taken from mucilage and surrounding seawater in the Sea of Marmara in April–May 2021. In another study, Danovaro et al. (2009) detected high concentrations of *E. coli* and coliform bacteria in mucilage samples from the Adriatic Sea coast in 2007, indicating the presence of pathogenic bacteria. It is not surprising that members of *Enterobacteriaceae* of faecal origin were detected at higher rates in the autumn (Figures 2 and 3), immediately following the mucilage disaster, and then decreased in the subsequent months.

In our study, September recorded the highest number of *L. monocytogenes* isolations compared to other months (Figures 2 and 3). *Listeria monocytogenes* is widespread in nature and can be found in various animals, mainly domestic farm animals. Especially aquatic environments have a significant potential for this dangerous bacterium to be transmitted to fish and shellfish and then to humans through the food chain (Kayode et al., 2021; Raschle et al., 2021). Changing climatic conditions and industrial activities may increase the likelihood of *Listeria* species in surface waters (El‐Shenawy & El‐Shenawy, 2006; Jami et al., 2014). In a study, the presence of *L. monocytogenes* in coastal waters has been associated with faecal contamination. The presence of this bacterium is crucial for assessing water quality in marine environments, particularly along the coasts of large cities. It also plays a key role in identifying the causes of pollution (El‐Shenawy & El‐Shenawy, 2006).

As seen in Figure 2, *V. parahaemolyticus* is the second most commonly isolated pathogen (78.4%) after *A. hydrophila* during the post‐mucilage fishing season. The high detection rate of *V. parahaemolyticus* in this study is consistent with the previous studies (Ali et al., 2021; Mok et al., 2021). In addition to high detection rates, *V. parahaemolyticus* was isolated from seafood samples collected in every month of the fishing season. *Vibrio cholerae*, the other pathogenic species of the *Vibrionaceae* family, was detected only in anchovy samples in October (Figures 2 and 3). The *Vibrio* species, whose main habitat is marine and estuarine water, are generally associated with aquatic animals (especially invertebrates) and plankton. Therefore, members of *Vibrio* commonly can be found in aquatic animals (Jones, 2014). Kokashvili et al. (2015) noted that the genus Vibrio includes pathogenic species such as *V. parahaemolyticus* and *V. cholerae*, which are important for public health. Studies conducted in various parts of the world have isolated these pathogens from coastal and marine waters, as well as from fish and shellfish (Jiang & Fu, 2001; Mok et al., 2021; Powell et al., 2013; Traoré et al., 2014). Mucilage, which is rich in phytoplankton (Fukao et al., 2009), is also a good habitat for *Vibrio* species. With the abundance of plankton, the probability of finding members of the *Vibrionaceae* family may increase (Asplund et al., 2011; Hsieh et al., 2007). Indeed, studies on the presence of pathogenic bacteria in marine environments particularly emphasize that *Vibrio* species are found in high quantities (Danovaro et al., 2009; Pistocchi et al., 2005). At the same time, Serratore et al. (1995) isolated many *Vibrio* species, including *V. parahaemolyticus*, from mucilaginous aggregates in the Northwest Adriatic Sea.

**FIGURE 4:**
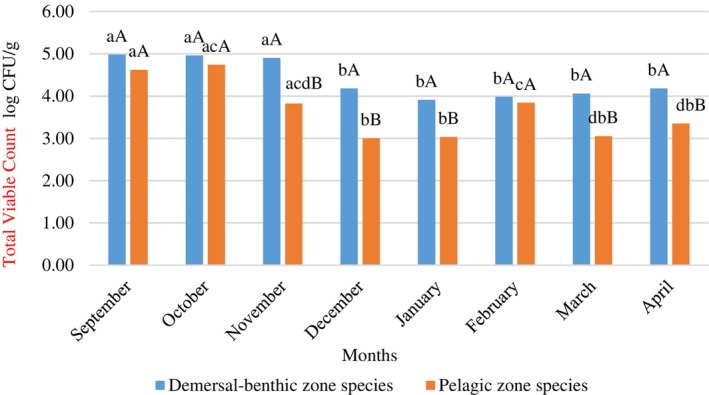
Monthly total viable count changes of demersal‐benthic zone species and pelagic zone species. ^a–d^: Lowercase indicate differences between months (*p* <0.05). ^A,B^: Uppercase indicate the differences between demersal‐benthic and pelagic zone species.

Following the mucilage event in the Sea of Marmara, *A. hydrophila* was detected in all seafood samples collected during the fishing season (Figures 2 and 4). The former monitoring studies also indicated a high isolation level and incidence of *A. hydrophila* in seafood (Dahdouh et al., 2016; Papadopoulou et al., 2007; Vivekanandhan et al., 2005). Altuğ et al. (2013) also reported that *A. hydrophila* was among the bacteria isolated from water samples collected from the Sea of Marmara between 2002 and 2011. *Aeromonas* spp., which are among the most significant inhabitants of various aquatic environments (Chen et al., 2021) and are considered emerging pathogens, have been reported to cause skin and soft tissue infections (Alkan‐Çeviker et al., 2019) as well as gastroenteritis (Greiner et al., 2021) in humans.

The mean total viable counts of all seafood species were significantly higher (*p* <0.05) in September, October and November compared to other months of the catching season (Table 1). One of the most important indicators used to determine microbial quality in foods is the total viable count (Üçok Alakavuk & Mol, 2021). The high total viable count in fish indicates anthropogenic pollution of their aquatic habitat or unhygienic practices during postharvest processes (Guiraud, 2003; Temiz, 1998). In this study, total *Enterobacteriaceae* counts also reached the highest level (*p* <0.05) in September compared to other months (Table 1). As the *Enterobacteriaceae* family includes both faecal and non‐faecal bacteria, elevated levels of *Enterobacteriaceae* in seafood suggest that the aquatic environment may be exposed to anthropogenic contamination or poor hygiene practices (Maloo et al., 2014; Temiz, 1998). Miranda and Zemelman (2001) stated that fish species living in waters close to urban sewage waters may be the carriers of *Enterobacteriaceae* species. *Clostridium perfringens* analysis was performed to indicate anaerobic microbiota and faecal contamination. The release of faecal pollution into surface waters constitutes environmental sources of faecal‐derived microorganisms, including pathogen *C. perfringens*. Bacteria used as indicators of faecal contamination, such as *E. coli* and Enterococci, have a limited lifespan in aquatic environments. This makes it challenging to assess the long‐term impact of pollution in both freshwater and marine systems. *Clostridium perfringens* has spore‐forming abilities that enable it to survive in harsh environments, facilitating long‐term detection in aquatic environments. This makes it a useful faecal indicator for aquatic environments (Hassan et al., 2015; Mueller‐Spitz et al., 2010). In this study, the highest value of *C. perfringens* was detected in October (1.71 log CFU/g) during the fishing season (Table 1). As the fishing season begins in autumn, shortly after the mucilage disaster, and while the seawater is still warm, the microbial load in seafood samples is higher compared to other seasons. Previous studies have shown that marine mucilage is a good reservoir for phytoplanktonic microorganisms, bacteria and viruses (Akcaalan et al., 2023; Danovaro et al., 2009; Del Negro et al., 2005; Ergul et al., 2021). Many studies have reported that high ambient temperatures increase both the number and diversity of microbiota (Hasan et al., 2023; Janelidze et al., 2011; Novoslavskij et al., 2016; Shinde et al., 2023). *Bacillus cereus*, which can survive under many adverse conditions thanks to its resistant endospores, proliferates across various environmental niches (Hsu et al., 2021). *Bacillus cereus* growth was not observed in any of the samples in this study.

**TABLE 1:** Total viable count, total *Enterobacteriaceae* count and *Clostridium perfringens* count results (i.e., mean of all species).

Months	Total viable count (log CFU/g)	Total *Enterobacteriaceae* count (log CFU/g)	*Clostridium perfringens* count (log CFU/g)
September	4.82 ± 1.05^a^	4.98 ± 1.45^a^	1.59 ± 0.83^a^
October	4.89 ± 0.88^a^	4.06 ± 0.90^b^	1.71 ± 1.02^ab^
November	4.57 ± 0.95^a^	3.81 ± 1.30^bc^	1.57 ± 1.05^bd^
December	3.86 ± 0.95^b^	3.21 ± 0.86^cd^	0.99 ± 0.00^c^
January	3.47 ± 1.04^b^	2.92 ± 0.94^d^	1.14 ± 0.42^cde^
February	3.91 ± 0.88^b^	3.12 ± 0.79^cd^	1.10 ± 0.36^cd^
March	3.69 ± 1.19^b^	3.00 ± 0.80^d^	1.05 ± 0.19^c^
April	3.76 ± 0.77^b^	3.37 ± 0.90^cd^	1.49 ± 0.53^abe^

*Note*: ^a–e^: Different lowercase in the same column indicate significant differences (*p* <0.05). ±: Standard deviation.

**FIGURE 5:**
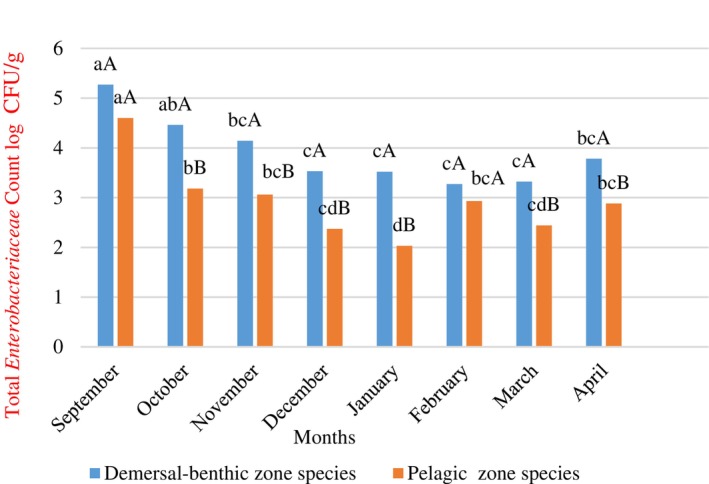
Monthly total *Enterobacteriaceae* count changes of demersal‐benthic zone species and pelagic zone species. ^a–d^: Lowercase indicate differences between months (*p* <0.05). ^A,B^: Uppercase indicate the differences between demersal‐benthic and pelagic zone species.

Monthly changes in TVC and TEC values, the two main indicators used to determine microbiological quality (Janelidze et al., 2011; Üçok Alakavuk & Mol, 2021), are shown in Figures 4 and 5. It was found that demersal‐benthic species contained significantly higher levels of these bacteria (*p* <0.05) than pelagic species. Additionally, bacterial levels were generally higher in autumn than in other seasons across all groups. Although the count of *Clostridium perfringens* showed some fluctuations between months, it remained below 2 log CFU/g throughout the fishing season.

## CONCLUSIONS

This study highlights the urgent need to address the universal calls for action put forward by the UN, aiming to solve global problems affecting humanity by 2030. Marine mucilage is an ongoing environmental issue, particularly in Mediterranean Basin countries, driven by economic activities and ecological impacts. Its primary causes include organic and industrial pollution, global warming and increasing nutrient loads in seas due to meteorological conditions that can trigger mucilage outbreaks. Permanent measures to combat the elevated nutrient load causing mucilage formation are essential. Key areas of focus include periodic monitoring of coastal water quality, effective management of water resources, and the sustainability of marine ecosystems. Local governments should enhance water quality in line with global sustainability goals by halving untreated wastewater discharge and significantly increasing recycling and safe reuse on a global scale. Priority should be given to strengthening infrastructure, promoting efficient resource use and encouraging the adoption of cleaner and more environmentally friendly technologies and industrial processes. Ensuring a healthy, high‐quality life should be a key local development goal. This aligns with the global aim to significantly reduce diseases and deaths caused by air, water and soil pollution from harmful chemicals. As the frequency of mucilage events increases worldwide, ensuring the safety of seafood for consumption and taking appropriate precautions become increasingly important.

Unlike previous studies, this research addresses both food safety and public health aspects of marine mucilage. The fishing season in the Sea of Marmara begins in the autumn, and in September, the diversity of pathogens was remarkably high. The proximity of this period to the mucilage disaster, along with elevated sea temperatures, contributed to these findings. The results clearly indicate that consuming seafood shortly after a mucilage event may pose risks to human health. Pathogenic microorganisms in sea mucilage may contaminate seafood, presenting a potential health risk upon consumption. It is crucial to inform the public about these potential dangers and raise awareness through media outlets. Ensuring food safety at every stage from production to consumption is vital. As the global food loss index continues to rise, environmental disasters such as mucilage pose a serious threat to food security. The findings also emphasize the importance of the One Health approach, which links public health to human, animal and environmental interactions.

Based on these findings, future research should focus on several key areas to further understand the impact of marine mucilage on seafood safety and public health. First, more comprehensive studies are needed to investigate the long‐term effects of mucilage on the microbial load of various seafood species beyond a single fishing season, to assess whether mucilage‐related contamination persists in marine environments and seafood over time. Additionally, future studies should expand the range of microbial pathogens tested, including non‐O157 STECs and *Vibrio vulnificus*, to provide a broader evaluation of potential risks to human health. Research should also explore the impact of mucilage on different aquatic ecosystems, particularly comparing its effects on pelagic versus benthic species, as significant differences in microbial contamination were observed between these groups.

Finally, future research should consider developing and testing potential intervention strategies, such as improved seafood depuration techniques or monitoring programs, to mitigate the risks associated with mucilage events. These findings are critical for informing public health policies and ensuring seafood safety in regions prone to mucilage outbreaks. This study's results align with several UN Sustainable Development Goals (SDGs), particularly Goal 3 (Good Health and Well‐being), Goal 12 (Responsible Consumption and Production) and Goal 14 (Life Below Water). It is crucial to manage chemicals and waste that contribute to mucilage formation throughout their life cycle, in line with international guidelines. Reducing their release into air, water and soil is necessary to minimize the negative impacts on human health and the environment.

## AUTHOR CONTRIBUTIONS

**Didem Üçok:** Investigation; writing – original draft; formal analysis; software; methodology; writing – review and editing; project administration; data curation; validation. **Şehnaz Yasemin Tosun:** Investigation; writing – original draft; project administration; formal analysis; methodology; validation; visualization. **Nuray Erkan:** Conceptualization; writing – original draft; funding acquisition; writing – review and editing; methodology; project administration; supervision; resources. **İdil Can Tunçelli:** Investigation; methodology; project administration; formal analysis; visualization. **Hande Doğruyol:** Investigation; methodology; formal analysis; project administration. **Şafak Ulusoy:** Formal analysis; project administration; methodology. **Sühendan Mol:** Resources; project administration. **Özkan Özden:** Resources; project administration. **Eda Dagsuyu:** Project administration. **Refiye Yanardag:** Project administration.

## CONFLICT OF INTEREST STATEMENT

The authors declare no conflicts of interest.

## Data Availability

The data that support the findings of this study are available from the corresponding author upon request.
